# Connecting the Dots Between Hypercholesterolemia and Alzheimer’s Disease: A Potential Mechanism Based on 27-Hydroxycholesterol

**DOI:** 10.3389/fnins.2022.842814

**Published:** 2022-04-07

**Authors:** Mingan Wu, Yingying Zhai, Xiaoyi Liang, Weichun Chen, Ruiyi Lin, Linlin Ma, Yi Huang, Di Zhao, Yong Liang, Wei Zhao, Jiansong Fang, Shuhuan Fang, Yunbo Chen, Qi Wang, Weirong Li

**Affiliations:** ^1^Science and Technology Innovation Center, Guangzhou University of Chinese Medicine, Guangzhou, China; ^2^Institute of Clinical Pharmacology, Guangzhou University of Chinese Medicine, Guangzhou, China

**Keywords:** Alzheimer’s disease, hypercholesterolemia, 27-hydroxycholesterol, pathogenesis, drug

## Abstract

Alzheimer’s disease (AD), the most common cause of dementia, is a complex and multifactorial disease involving genetic and environmental factors, with hypercholesterolemia considered as one of the risk factors. Numerous epidemiological studies have reported a positive association between AD and serum cholesterol levels, and experimental studies also provide evidence that elevated cholesterol levels accelerate AD pathology. However, the underlying mechanism of hypercholesterolemia accelerating AD pathogenesis is not clear. Here, we review the metabolism of cholesterol in the brain and focus on the role of oxysterols, aiming to reveal the link between hypercholesterolemia and AD. 27-hydroxycholesterol (27-OHC) is the major peripheral oxysterol that flows into the brain, and it affects β-amyloid (Aβ) production and elimination as well as influencing other pathogenic mechanisms of AD. Although the potential link between hypercholesterolemia and AD is well established, cholesterol-lowering drugs show mixed results in improving cognitive function. Nevertheless, drugs that target cholesterol exocytosis and conversion show benefits in improving AD pathology. Herbs and natural compounds with cholesterol-lowering properties also have a potential role in ameliorating cognition. Collectively, hypercholesterolemia is a causative risk factor for AD, and 27-OHC is likely a potential mechanism for hypercholesterolemia to promote AD pathology. Drugs that regulate cholesterol metabolism are probably beneficial for AD, but more research is needed to unravel the mechanisms involved in 27-OHC, which may lead to new therapeutic strategies for AD.

## Introduction

Alzheimer’s disease (AD), the most common cause of dementia, is a pervasive, progressive neurodegenerative disease characterized by cognitive impairment. With more than 55 million people in the world currently affected by dementia, AD is recognized as a rising global health crisis (Alzheimer’s Disease International and McGill University, 2021). β-amyloid (Aβ) plaques and neurofibrillary tangles (NFT) are generally considered to be the cardinal pathological features of AD (Busche and Hyman, 2020). Researcher hopes are high that eliminating pathology could lead to revolutionary treatment for AD and are committed to developing drugs that target Aβ and tau. However, they are mostly left empty-handed and new directions for AD research are urgently required. AD is a multifactorial and complex disease caused by a combination of genetic and environmental risk factors. In recent years, risk factor management has been proposed as an effective way to slow down AD development. Modifiable risk factors present in midlife such as hypertension (Lennon et al., 2019), diabetes (González et al., 2020), and hypercholesterolemia (Anstey et al., 2008) are associated with cognitive decline in later life (Gottesman et al., 2017; Knopman et al., 2018). It is important to note that risk factors do not occur alone, but often coexist and interact with each other. For instance, it is well known that hypercholesterolemia is a major risk factor for atherosclerosis. In the Rotterdam study, a correlation was found between atherosclerosis and AD (Hofman et al., 1997). Furthermore, hypercholesterolemia may induce hypertension because it increases the secretion of vasoconstrictor molecules and decreases the bioavailability of nitric oxide (Sposito, 2004). Hypertension can lead to pathological changes such as Aβ plaques and tau tangles, either in humans or in animal models (Lennon et al., 2021). Of note, this review excludes the indirect effects of hypercholesterolemia on AD processes by inducing other diseases, but focuses more on the role of hypercholesterolemia itself on AD. Hypercholesterolemia is defined as high plasma cholesterol levels with normal plasma triglycerides (Martinez-Hervas and Ascaso, 2019). There are two main sources of cholesterol in the human body: about 70% of cholesterol is synthesized by the body, while the other 30% comes from dietary intake (Kapourchali et al., 2016). Cholesterol is widely present in all tissues, with about 1/4 of it distributed in the brain. As an important lipid class, Brain cholesterol exists mainly in the unesterified form, with one-third of the cholesterol present in cellular membranes and most in myelin sheaths (Karasinska and Hayden, 2011). For neurons and astrocytes, cholesterol plays an important role in maintaining the structural integrity of the plasma membrane and regulating its fluidity (Dietschy and Turley, 2004). Cholesterol is also critical for forming myelin sheaths in oligodendrocytes, which provides electrical insulation around axons to speed up the propagation of electrical signals through the nervous system (Saher and Simons, 2010). Demyelination has become a biomarker for dementia pathology (Bouhrara et al., 2018). Moreover, cholesterol is required for synapse and dendrite formation (Goritz et al., 2005). Cholesterol enhances presynaptic differentiation, which is vital for continuous synaptogenesis and important for the stability of neurotransmitters (Dai et al., 2021). In addition, cholesterol is a component of lipid rafts, which perform roles in signal transduction, cell adhesion, and lipid/protein sorting (Kao et al., 2020). Of note, lipid rafts contain AD-related proteins such as APP, BACE1, and γ-secretase (El Gaamouch et al., 2016). Additionally, lipid rafts provide a platform for Aβ to interact with ApoE (Kawarabayashi et al., 2004). The common variants of ApoE are AopE2, AopE3, and AopE4, with ApoE4 serving as the strongest genetic risk factor for sporadic AD (Kunkle et al., 2019). Substantial evidence suggests that ApoE4 promotes Aβ aggregation and deposition in the brain (DeMattos et al., 2004; Dolev and Michaelson, 2004). Cholesterol metabolism plays an essential role in maintaining brain function, whereas cholesterol dysregulation serves as a potential risk factor for diseases, including AD (Di Paolo and Kim, 2011). Interestingly, there is a near-perfect regulatory system in the brain that prevents serum cholesterol from entering the central nervous system (CNS) to maintain stable cholesterol levels and support normal brain function (Gliozzi et al., 2021). Thus, it remains to be explained how hypercholesterolemia affects AD pathogenesis. Herein, we reviewed the potential mechanisms of hypercholesterolemia with AD, aiming to provide new strategies for preventing or delaying the onset of AD.

## Evidence for the Role of Hypercholesterolemia in Alzheimer’s Disease

In the last decade, the relationship between cholesterol and AD has been thoroughly investigated in epidemiological studies. Scholars screened 17 studies for a meta-analysis and showed that adults with high total cholesterol (TC) in midlife are at higher risk for AD (Anstey et al., 2017). Similar results were seen in a longitudinal, population-based prospective cohort study, which followed participants without dementia for 13 years and found that higher TC concentrations at baseline were associated with an increased risk of AD (Schilling et al., 2017). In a United States-based cohort analysis, higher TC and triglyceride levels in midlife were associated with a greater 20-year decline in memory, as judged by decreased scores on tests of executive functioning, processing speed, and sustained attention (Power et al., 2018). In China, participants from the cohort study “the Effects and Mechanism Investigation of Cholesterol and Oxysterol on Alzheimer’s disease” completed cognitive tests and lipid level assessment. The data suggest that increased serum TC is significantly associated with accelerated cognitive decline (An et al., 2019). Additionally, elevated TC concentrations in late life can also accelerate cognitive decline, as reported in two studies in China (Ma et al., 2017; Guo et al., 2020). However, other different studies have reported that cholesterol intake and peripheral cholesterol levels are not associated with an increased risk of AD (Tan et al., 2003; Ylilauri et al., 2017). Another study suggests that elevated TC levels reduce the risk for dementia in older adults (Ding et al., 2021). Epidemiological investigations have shown conflicting results on the role of hypercholesterolemia for AD, which highlights the complex role of serum cholesterol in cognitive decline. Interestingly, experimental studies have shown that a high cholesterol diet (HCD), to some extent, promotes AD development. A previous study showed that Japanese white rabbits on HCD were found to have changes in brain metabolism and structure that were similar to those of human AD (Jin et al., 2018). In rats and mice, HCD also induces significant cognitive impairment and Alzheimer’s-like disease (Abo El-Khair et al., 2014; Ledreux et al., 2016; Mancini et al., 2018; Ledo et al., 2019; Mancini et al., 2021). Altogether, hypercholesterolemia is likely to promote AD development as a risk factor, especially with elevated TC levels in midlife.

## Correlation of Hypercholesterolemia With Alzheimer’s Disease Pathogenesis

### Hypercholesterolemia and Amyloid Accumulation

The abnormal accumulation of Aβ is one of the pathological hallmarks of AD (Hardy and Higgins, 1992). Aβ is produced when amyloid precursor protein (APP) is cleaved via the amyloidogenic pathway, which is mediated by β-secretase (BACE) and γ-secretase (Vassar et al., 1999). γ-secretase is a membrane protease complex that contains presenilin as the catalytic subunit. Of note, APP can also be cleaved via the non-amyloid pathway, a process mediated by α and γ secretase, which do not produce Aβ (Dar and Glazner, 2020). As aducanumab receives U.S. Food and Drug Administration approval, it signals that anti-amyloid immunotherapy will provide the first therapy to slow or reverse the progression of AD (U.S. Food and Drug Administration, 2021). Although opponents of the amyloid hypothesis argue that Aβ is an epiphenomenon and that the true cause of the disease remains unclear. Nearly indisputable human genetic data, as well as extensive evidence from animal models, suggest a key role for Aβ in AD (Musiek et al., 2021). Positron emission tomography (PET) is an important tool for quantifying brain amyloidosis in patients with suspected AD. In a longitudinal comparison study, amyloid PET scan data derived from 207 non-demented participants in the Alzheimer’s Disease Neuroimaging Initiative (ADNI) were analyzed in conjunction with baseline cholesterol. As expected, the results showed that higher serum cholesterol levels accelerate Aβ deposition in the brain (Souza et al., 2020). High-field magnetic resonance imaging is another technology used for Aβ visualization. When such a technique was applied to detect cholesterol-fed rabbits, signal voids were observed in the brain, which corresponded to Aβ-positive plaques (Chen Y. X. et al., 2018). In addition, the apolipoprotein E (ApoE) knock-out mice were treated with a high-fat diet and injected intracerebroventricularly with Aβ_25–35_, the results showed that hypercholesterolemia accelerated Aβ accumulation and tau pathology, which subsequently deteriorated cognitive impairment (Park et al., 2013). Similar results were obtained in several different strains of mice and Sprague-Dawley rats, with diet-induced hypercholesterolemia accelerating Aβ accumulation in the brain (Shie et al., 2002; Li et al., 2003; Umeda et al., 2012; Liu et al., 2018). Various animal studies suggest that hypercholesterolemia increases Aβ by affecting APP processing *in vivo* (Refolo et al., 2000; Ullrich et al., 2010). Although Aβ and APP levels were not affected in the early stages of hypercholesterolemia, the levels of presenilin 1 (PS1) that initiates Aβ production were increased (Chen et al., 2016). Collectively, the accelerated accumulation of Aβ by hypercholesterolemia is likely to be a major cause of cognitive decline.

### Hypercholesterolemia and Tau Pathology

Tau is a microtubule-associated protein mainly expressed in neurons, and one of its main functions is to maintain the stability of axonal microtubules (Naseri et al., 2019). Hyperphosphorylation may disengage tau from microtubules, and tau tends to misfold and aggregate to form NFT, which eventually impairs neuronal function. In addition, pathological tau induces synaptic dysfunction, which is another early pathological manifestation of AD (Wu et al., 2021). Researchers found a significant association between hypercholesterolemia and all AD neuropathological outcomes, including NFT, by analyzing neuropathological and clinical data from subjects in the National Alzheimer’s Disease Coordinating Center (NACC) (Xu et al., 2020). In animal experiment, a high-fat/cholesterol diet alters insulin/IGF signaling in C57BL/6 mice, which increases hippocampal hyperphosphorylated tau levels and leads to AD-like cognitive impairment (Bhat and Thirumangalakudi, 2013). Phosphorylated tau expression was also increased in ApoE knock-out mice on a high-fat diet with intracerebroventricular injections of Aβ _25–35_ (Park et al., 2013). In rats treated with a high-fat/cholesterol diet, phosphorylated tau levels increased similarly (Abd Al Haleem and El-Bakly, 2019). However, not all studies have come to the same conclusion. In humanized tau transgenic mice, HCD is not sufficient to induce tau pathology, either tau phosphorylation or aggregation (Gratuze et al., 2016). The discrepancy may be due to differences in animal strains, which have different responses to HCD. Overall, additional studies are needed to fully understand the relationship between hypercholesterolemia and tau pathology to provide a definitive answer.

### Hypercholesterolemia and Neuroinflammation

In addition to Aβ and NFT, neuroinflammation also holds a prominent role in the pathogenesis of AD (Leng and Edison, 2021). Neuroinflammation is an inflammatory response that occurs within the CNS, with microglia and astrocyte involved in the process. Microglia accelerate AD pathogenesis by releasing inflammatory mediators but play a beneficial role in amyloid plaque clearance (Cai et al., 2014). Activated microglia produces and secretes several proinflammatory mediators, such as interleukin-1β (IL-1β), IL-6, IL-18 and tumor necrosis factor (TNF) (Mandrekar-Colucci and Landreth, 2010; Leng and Edison, 2021). The release of pro-inflammatory molecules causes synaptic dysfunction, neuronal death and inhibition of neurogenesis (Lyman et al., 2014). In AD patients, there is a positive correlation between microglia activation and tau aggregation, as well as amyloid deposition (Dani et al., 2018). Astrocytes have A1 phenotype and A2 phenotype. The A1 astrocyte phenotype is involved in neuroinflammation through the nuclear factor-κB (NF-κB) pathway that releases inflammatory cytokines (Leng and Edison, 2021). Exposure of astrocytes to Aβ would release cytokines, ILs, NO and other potentially neurotoxic mediators (Ahmad et al., 2019). In animal studies, both 4-month-old C57BL/6 and LDL receptor-deficient mice developed cognitive dysfunction on a high fat/cholesterol diet for 8 weeks, with neuroinflammation thought to play a primary role. It is because of microglia and astrocytes that have been activated in mice hippocampus, with increased expression of cytokines and mediators, which include TNF-a, IL-1β, IL-6, nitric oxide synthase 2, and cyclooxygenase 2 (Thirumangalakudi et al., 2008). Similarly, Wistar rats on a high-fat/cholesterol diet had significantly elevated IL-1β, IL-2, IL-6 and TNF-α levels in the hippocampus (Abuelezz and Hendawy, 2021). However, on 6-month-old CD1 mice, HCD for 8 weeks did not show microglia activation in the hippocampus and only mild astrogliosis was observed. In 16-month-old CD1 mice, HCD enhanced microglia activation with an increase in IL-1β, IL-6 and TNF-α expression, however, HCD also promoted microglia polarization to M2-like phenotype, which is characterized by secretion of anti-inflammatory cytokines, such as IL-4 and IL-10(Chen Y. et al., 2018).

### Hypercholesterolemia and Oxidative Stress

Oxidative stress is an imbalance between the oxidative and antioxidant systems favoring the oxidative system, and its contribution to AD progression has been demonstrated in a wide range of studies (Butterfield and Halliwell, 2019). Reactive oxygen species (ROS) and reactive nitrogen species (RNS) are the most representative oxidants that have significant effects on redox biology and cause oxidative stress (Ezraty et al., 2017). Unlike oxidants, the antioxidant system is mainly served by enzymes, which mainly include superoxide dismutase (SOD), glutathione peroxidase (GPX), and others. SOD is an endogenous enzyme that converts superoxide radicals into diatomic oxygen and hydrogen peroxide. Hydrogen peroxide is further converted to water by GPX. In addition, glutathione (GSH) is the main non-enzymatic antioxidant that scavenges ROS to protect the body from oxidative stress damage. The major biomarkers of oxidative stress include malondialdehyde, protein carbonyls and so on (Sies et al., 2017). Earlier studies have shown that HCD increases malondialdehyde level and thiobarbituric acid-reactive substances (TBARS) level in Wistar and Sprague-Dawley rats’ brains (Gökkus̨u and Mostafazadeh, 2003; Montilla et al., 2006; Otunola et al., 2014; Hegazy et al., 2016). Malondialdehyde is one of the lipid peroxidation products, and TBARS is considered the end product of malondialdehyde. Moreover, protein carbonyl levels were increased in the hippocampus of albino rabbits fed with HCD (Aytan et al., 2008). In addition to causing lipid peroxidation, HCD decreases antioxidant enzyme activity in the brain. In Wistar rats, HCD decreased SOD, GPX and GSH activity in the brain (Montilla et al., 2006; Afonso et al., 2013; Otunola et al., 2014). GSH is the body’s master antioxidant that prevents ROS accumulation. Similarly, HCD decreased GSH levels in both Sprague-Dawley rats and LDLR -/- mice (Gökkus̨u and Mostafazadeh, 2003; de Oliveira et al., 2013).

### Hypercholesterolemia and Blood-Brain Barrier Breakdown

The blood-brain barrier (BBB) is a highly selective semipermeable cellular border that regulates the transport of substances into and out of the CNS, which is essential for proper neuronal functioning (Cai et al., 2018). BBB breakdown allows neurotoxic substances to enter the brain and harm neurons, and/or trigger amyloid deposits, which accelerate the course of AD (Sweeney et al., 2018). Nowadays, BBB breakdown has been identified as an early biomarker of human cognitive dysfunction independent of Aβ and tau (Nation et al., 2019). In rabbits fed on HCD, BBB tight junction proteins expression was down-regulation and IgG was increased in the hippocampus, suggesting that BBB is disrupted because IgG is not present in normal brain parenchyma (Chen et al., 2008). BBB permeability increase induced by HCD can be ameliorated by simvastatin, a medicine used to lower cholesterol (Jiang et al., 2012). In wild-type and LDLR−/− mice, BBB disruption is reflected in increased permeability to sodium fluorescein in the hippocampus and decreased levels of claudin-5 and occludin mRNA (de Oliveira et al., 2020). Furthermore, cholesterol supplementation in rats during hypertension (Kalayci et al., 2009), or diabetes combined with hypercholesterolemia (Acharya et al., 2013) also increased the permeability of the BBB. In hypercholesterolemic patients, increased BBB permeability was demonstrated by quantifying the water exchange rate across the BBB (Shao et al., 2019). Overall BBB disruption appears to be a relevant event for hypercholesterolemia-induced changes in the brain. However, the mechanism by which hypercholesterolemia increases the permeability of the BBB remains to be elucidated. Current evidence has shown that a cholesterol-rich diet increases plasma cholesterol concentrations and positively correlates with AD pathology. As serum cholesterol is blocked from entering the brain, it is necessary to review cholesterol metabolism in the brain, which will be key to elucidating the potential link between hypercholesterolemia and AD.

## Cholesterol Homeostasis in the Brain

### Cholesterol Metabolism in Central Nervous System

The brain is an organ rich in cholesterol, accounting for approximately 25% of total body cholesterol, which is higher than any other organ (Björkhem and Meaney, 2004).

**FIGURE 1:**
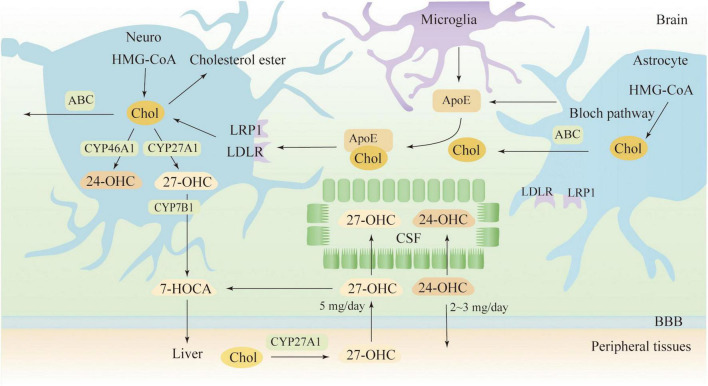
Cholesterol metabolism in the brain. 7-HOCA, 7α-hydroxy-3-oxo-4-cholestenoic acid; 24-OHC, 24(S)-hydroxycholesterol; 27-OHC, 27-hydroxycholesterol; ABC, ATP-binding cassette; ApoE, apolipoprotein E; Chol, cholesterol; CSF, cerebrospinal fluid; LDLR, low-density lipoprotein receptor; and LRP1, LDL receptor-like protein 1.

As BBB prevents the direct uptake of cholesterol, most of the cholesterol in the brain comes from its synthesis, a complex process involving multiple enzymatic reactions (Genaro-Mattos et al., 2019). In the CNS, it is believed that neurons synthesize only a limited amount of cholesterol via the Kandutsch-Russell pathway, and more sources of cholesterol are dependent on astrocytes, which synthesize cholesterol via the Bloch pathway (Pfrieger and Ungerer, 2011; Zhang and Liu, 2015). A recent study indicates that both neurons and astrocytes preferentially synthesize cholesterol via the Bloch pathway and that endogenous cholesterol accumulates in neurons over time (Genaro-Mattos et al., 2019). As shown in Figure 1, neurons take up cholesterol from astrocytes mainly via ApoE transport. Most ApoE is synthesized by astrocytes and microglia in the CNS, and under normal physiological conditions, neurons do not produce ApoE (Vance and Hayashi, 2010). ApoE binds to cholesterol for transport and releases cholesterol to neurons by binding to the LDLR and LDLR-related protein 1 (LRP1) receptors (Petrov and Pikuleva, 2019). LDLR and LRP1 are expressed by neurons and glial cells, but LDLR is mainly expressed in glial cells, while LRP1 is mainly expressed by neurons. Other receptors in the brain are also involved in cholesterol transport, such as VLDLR, ApoER2/LRP8, LRP4/MEGF7, LRP1B, megalin/LRP2, LRP5, LRP6, and SorLA/LR11(Jin et al., 2019). To maintain a steady state, cholesterol in the brain can be converted to 24 (S)−hydroxycholesterol (24-OHC) by cytochrome P450 46A1 (CYP46A1), a process that is completed only in neurons (Lund et al., 1999). 24-OHC crosses the BBB into the peripheral circulation more readily than cholesterol and is subsequently picked up by plasma lipoproteins and transported to the liver for metabolism (Bjorkhem et al., 2001). The liver has several mechanisms to eliminate 24-OHC, such as being excreted directly as a prototype or its conjugated form, or being hydroxylated and excreted, as well as being converted to cholic or chenodeoxycholic acid (Bjorkhem et al., 2001). Cholesterol can also be excreted from neurons via ATP-binding cassette (ABC) transporters, such as ABCA1, ABCG1 and ABCG4 (Kim et al., 2008). Subsequent cholesterol transport is mediated by ApoE, but it seems less important than the 24-OHC-mediated mechanism (Björkhem et al., 1998). Surplus cholesterol is converted to cholesteryl esters for storage by Acyl-CoA cholesterol acyltransferase (ACAT) (Leon et al., 2005). Serum 24-OHC levels indicate a disturbance in brain cholesterol turnover, as 24-OHC primarily comes from the brain. 24-OHC has been reported to be exported from the brain at a rate of about 2-3 mg/day (Iuliano et al., 2015). Similarly, peripheral cholesterol can also enter the CNS in the form of side-chain oxidation, and 27-OHC is the most abundant oxysterol in blood circulation. The formation of 27-OHC is mediated by cytochrome P450 family 27 subfamily A member 1 (CYP27A1), which introduces the hydroxyl groups at position 27, a process that occurs in almost all cells *in vivo* but is synthesized at low rates in neurons and glial cells (Russell, 2000). A previous study suggests that approximately 5 mg of 27-OHC per day flows into the brain and that more 27-OHC will enter the brain if the integrity and function of the BBB is impaired (Heverin et al., 2005; Björkhem et al., 2009). Generally, the flux of 27-OHC into the brain is higher in males than females, but oxygen steroid levels are not affected by sex differences (Parrado-Fernandez et al., 2021). In neuronal cells, 27-OHC will be metabolized to 7a-hydroxy-3-oxo-4-cholestenoic acid (7-HOCA) by the action of the CYP7B1 enzyme (Meaney et al., 2007). 27-OHC enters the brain through free diffusion, so there is a correlation between the level of 27-OHC in the cerebrospinal fluid (CSF) and the level of 27-OHC in the circulation. Although HCD did not alter brain cholesterol levels in animal models, however, fluxes of 27-OHC into the brain were significantly increased (Marwarha and Ghribi, 2015). In addition to 24-OHC and 27-OHC, small amounts of other oxysterols were found in the brain, which includes 7α-hydroxycholesterol, 7β-hydroxycholesterol, 4β-hydroxycholesterol, α-epoxide, β-epoxide and 7-ketocholesterol, among others (Hascalovici et al., 2009). Cholesterol can also be metabolized to 25-hydroxycholesterol, which promotes IL-1β-mediated neuroinflammation and is involved in AD pathogenesis (Wong et al., 2020). Generally, 24-OHC and 27-OHC are considered more closely associated with AD pathogenesis, although several types of cholesterol metabolites are present in human brain and their levels will be altered in pathological states (Gamba et al., 2015).

### Twenty Four-OHC and 27-OHC in Brains With and Without Alzheimer’s Disease

Oxidized cholesterols play an important role in maintaining cholesterol homeostasis and its misregulation in the CNS has been reported to be associated with neurodegeneration. An earlier study had reported a decrease in 24-OHC and an increase in 27-OHC in brain samples from AD patients (Heverin et al., 2004). More specifically, such changes occurred at advanced stages of the AD, with CYP46A1 and CYP27A1 mRNA levels significantly decreased and increased in the AD brain, respectively (Testa et al., 2016). In the CSF, both 24-OHC and 27-OHC levels were significantly higher in those diagnosed with early AD than in controls (Wang et al., 2016). Similar finding has been reported in AD patients’ plasma (Zarrouk et al., 2020). The altered 24-OHC levels in CSF are thought to be caused by neuronal damage and/or demyelination, while elevated 27-OHC levels are attributed to dysfunction of BBB and blood-CSF barrier (Leoni et al., 2006). Recent study results revealed that not only 24-OHC but also 24-OHC/27-OHC ratio was higher in subjects with AD pathology (Jahn et al., 2021). Due to the reduction of CYP46A1 in AD brain, single nucleotide polymorphisms in CYP46A1 have been studied, in which CYP46A1 introns 1 (rs7157609) and 3 (rs4900442) were found to increase the risk of AD (Kölsch et al., 2009). Additionally, CYP46A1 intron 2 (rs754203) has a synergistic effect with APOE4 on increasing AD risk (Borroni et al., 2004; Li et al., 2006). A study conducted in Finland found a higher frequency of rs754203 CC genotypes than CT and TT genotypes in AD patients with onset over 65 years of age (Helisalmi et al., 2006). In patients with AD in China, the frequency of at least one CYP46A1 T allele (C/T or T/T) was higher (He et al., 2012). However, other studies found that rs754203 (Wang and Jia, 2007), rs4900442 (Li et al., 2006) are not associated with AD. Overall, CYP46A1 polymorphisms affect AD risk, but these contradictory reports need further clarification (Li et al., 2018). CYP46A1 maintains the balance of cholesterol metabolism in the brain by catalyzing the conversion of cholesterol to 24-OHC. Of note, 27-OHC produced by CYP27A1 also affects cholesterol homeostasis in the brain. 27-OHC is mainly derived from the peripheral circulation and is likely to be a bridge between hypercholesterolemia and AD. A case-control study showed a significant association between high plasma levels of 27-OHC and mild cognitive impairment (Liu et al., 2016). Another randomized controlled trial suggests that reducing serum 27-OHC levels by managing lifestyle and vascular factors is beneficial for improving cognitive function (Sandebring-Matton et al., 2021). In experimental studies, HCD upregulates CYP27A1 expression and increases plasma 27-OHC levels in rats, thereby affecting peripheral cholesterol metabolism (Zhang et al., 2018). Further, 27-OHC negatively affects cognitive function and cholesterol metabolism in rats when it is injected into the body through the tail vein (Zhang et al., 2015). Additionally, cholesterol diet affected spatial learning in wild-type mice but not in Cyp27KO mice lacking 27-OHC (Heverin et al., 2015).

## Mechanisms of 27-OHC Affecting AD Pathogenesis

### Effect on Aβ

**FIGURE 2:**
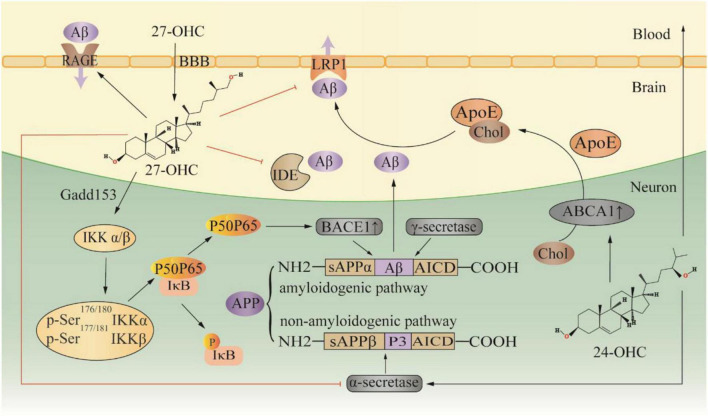
Effect of 27-OHC on Aβ production and elimination. 24-OHC, 24 (S) -hydroxycholesterol; 27-OHC, 27-hydroxycholesterol; ABCA1, ATP-binding cassette A1; ApoE, apolipoprotein E; APP, amyloid precursor protein; BBB, blood-brain barrier; Chol, cholesterol; Gadd153, growth arrest and DNA damage-induced gene 153; IDE, insulin-degrading enzyme; LRP1, LDL receptor-like protein 1; and RAGE, receptor for advanced glycation end products.

Twenty seven-OHC increases the accumulation and deposition of Aβ in the brain by regulating the metabolic processes of Aβ, including the production, transportation, and elimination (Figure 2). In APP/PS1 mice, subcutaneous injection of 27-OHC increased gene and protein expression of APP, BACE1, and receptor for advanced glycation end products (RAGE), while decreasing expression of a disintegrin and metalloprotease 10, LRP1, and insulin-degrading enzyme (IDE) (Zhang et al., 2019). Substantial evidence suggests that activation of the NF-κB signaling pathway increases BACE1 expression, which promotes Aβ production (Shi et al., 2017; Kim et al., 2019; Ma et al., 2020; Zheng et al., 2020). In SH-SY5Y cells, 27-OHC evokes phosphorylation of IκB kinase complex by activating gadd153 (growth arrest and DNA damage-induced gene 153), which in turn causes IκB phosphorylation, and consequently leading to IκB degradation and NF-κB activation (Marwarha et al., 2013). The IκB degradation product, p65-p50 dimer, translocates to the nucleus and binds to the κB site in the BACE1 promoter region, thereby upregulating BACE1 expression (Marwarha et al., 2013). Silencing the gadd153 gene can reduce 27-OHC induced Aβ production and decreased APP and BACE1 levels (Prasanthi et al., 2011). Aβ is cleared from the brain by two major pathways: efflux through the BBB and degradation by proteases. LRP1 and RAGE are the main receptors for Aβ transport, which mediate Aβ efflux and influx into the brain, respectively (Wang et al., 2021). Additionally, IDE is one of the main peptidases involved in Aβ degradation, and 27-OHC treatment in mice resulted in a reduction of IDE in the brain (Marwarha et al., 2010; Zhang et al., 2019). Moreover, 27-OHC competitively inhibits the benefits of 24-OHC because of their similar structure. SH-SY5Y cells exposed to 24-OHC showed an increase in α-secretase activity, implying that 24-OHC promotes APP processing by activating the non-amyloid production pathway (Famer et al., 2007). Increased expression of ABCA1 is another benefit of 24-OHC (Prasanthi et al., 2009). ABCA1-mediated ApoE lipidation plays a key role in facilitating extracellular Aβ degradation by IDE and facilitating Aβ transport (Fan et al., 2009). Gene overexpression of ABCA1 can reduce Aβ and plaque (Wahrle et al., 2008). In contrast, gene inactivation of ABCA1 increased the level of Aβ and plaque pathology in a mouse model (Corona et al., 2016). In the CNS, liver X receptor (LXR), retinoid X receptor (RXR) and peroxisome proliferator-activated receptor (PPAR) regulate ABCA1 transcription and ApoE expression (Koldamova et al., 2014). 24-OHC and 27-OHC are endogenous activators of LXR. In human primary neurons, 27-OHC acts as an LXR ligand and activates ABCA1 to reduce extracellular Aβ levels (Kim et al., 2009). Of note, 24-OHC is a full agonist while 27-OHC is a partial agonist (Mutemberezi et al., 2016). Apparently, 24-OHC and 27-OHC compete for a finite number of binding sites, and 27-OHC will act as an antagonist.

### Other Mechanisms of 27-OHC Affecting AD Pathogenesis

In hTau-ApoE^+/+^ mice, a mouse expressing P301L mutant human tau as well as wild-type ApoE, HCD increased the number of neurons with hyperphosphorylated tau, which was associated with elevated 27-OHC levels (Glöckner et al., 2011). In hippocampal slices from New Zealand white rabbits, 27-OHC treatment increased tau phosphorylation by altering leptin signaling, a process that can be reversed by leptin supplementation (Marwarha et al., 2010). Further study revealed that the 27-OHC reduced leptin expression in human neuroblastoma SH-SY5Y cells by inducing endoplasmic reticulum stress that activates C/EBP homologous protein, which has a negative regulation on C/EBPα, a transcription factor necessary for leptin expression (Marwarha et al., 2012). Neuroinflammation also plays a role in the pathogenesis of AD. In AD patients, pro-inflammatory factors such as IL-1β, IL-6 and IL-8 were significantly increased, which coincided with changes in oxysterol levels such as 27-OHC (Testa et al., 2016). Similarly, an experimental study showed that 27-OHC increased TNF-α and IL-17 levels in the brain by subcutaneous injection in C57BL/6J mice (Wang et al., 2020). In SH-SY5Y cells, 27-OHC increased TNF-α and inducible nitric oxide synthase expression and decreased IL-10 levels by activating the TGF-β/NF-κB signaling pathway. For rat glioma cells, 27-OHC induces inflammatory damage by activating TLR4/TGF-β signaling (Ma et al., 2019). In addition, 27-OHC mediates RAGE upregulation in astrocytes and neurons via RXRγ receptor (Loera-Valencia et al., 2021a). RAGE activation is involved in the inflammatory response and promotes neurodegeneration (Yan et al., 2009). In terms of oxidative stress, 27-OHC not only increased ROS in astrocytes but also decreased the activity of antioxidant enzymes by regulating the Nrf2 pathway, which includes SOD, GSH and GPX (Ma et al., 2015). In addition to being directly related to pathogenesis, 27-OHC is also closely associated with AD-associated events. Synaptic dysfunction is a major feature of many neurodegenerative diseases, including AD (Jackson et al., 2019). Synaptic plasticity occurs in neural circuits during long-term memory formation (Zhou et al., 2021). Long-term potentiation (LTP) is one of the main forms of synaptic plasticity, and high-frequency stimulation-induced LTP is dependent on estrogen receptor (ER) activation (Tozzi et al., 2019). 27-OHC is an endogenous selective estrogen receptor modulator (He and Nelson, 2017), which may decrease LTP amplitude. Lower LTP responses are often seen in AD models, but not in all. In Cyp27Tg transgenic mice, 27-OHC enhances LTP at Schaffer collateral-CA1 synapses, which may be related to abnormally large dendritic spines in the stratum radiatum (Loera-Valencia et al., 2021b). It is important to note that LTP responses deviating from normal, either higher or lower, may lead to hippocampal circuit dysfunction. Additionally, oxysterol mixtures containing 27-OHC decreased neuronal postsynaptic density protein 95 (PSD95) levels, which suggests that oxysterols induce synaptotoxicity (Staurenghi et al., 2021). In New Zealand White rabbits, HCD increased 27-OHC levels in the brain, and 27-OHC decreases synaptic marker protein PSD-95 expression by downregulating ERα (Brooks et al., 2017). 27-OHC also decreased PSD95 levels in hippocampal rat primary neurons, however, it was attributed to REST-miR124a-PTBP1 axis dysregulation (Merino-Serrais et al., 2019). Dysfunction in the metabolism of glucose also leads to AD and mild cognitive impairment (Gamba et al., 2019). Excess 27-OHC reduces brain glucose uptake in CYP27A1 overexpressing mice fed with HCD. On the one hand, 27-OHC increased aminopeptidase A(AP-A) expression via LXR, and AP-A increased angiotensin III production, which inhibited glucose transporter type 4 (GLUT4) expression. On the other hand, 27-OHC increased aminopeptidase N (AP-N) expression and AP-N enhanced the degradation of angiotensin IV, a metabolite that activates GLUT4.

## Can Cholesterol Regulation Prevent or Delay AD?

### Cholesterol-Lowering Drugs

Statins are the most common cholesterol-lowering drugs, blocking cholesterol synthesis in the hepatic pathway by competitively inhibiting HMG-CoA reductase (Maron et al., 2000). Statins can be classified as hydrophilic or lipophilic according to their ability to dissolve in lipid media or water. Hydrophilic statins, such as pravastatin and rosuvastatin, are unable to enter tissues other than the liver. In contrast, lipophilic statins, such as simvastatin and atorvastatin, are more likely to penetrate the BBB (Climent et al., 2021). Pravastatin reduce the absolute levels of plasma cholesterol and 27-OHC in men, with a slight increase in the ratio of 27-OHC to cholesterol (Thelen et al., 2006b). Atorvastatin and simvastatin can also reduce plasma cholesterol and 27-OHC concentrations in normal subjects, but do not alter the ratio of 27-OHC to cholesterol (Thelen et al., 2006a). Results from a retrospective case-control study conducted in Germany showed a negative association between the use of statins and all-cause dementia including AD. These statins included pravastatin, rosuvastatin, atorvastatin, fluvastatin, lovastatin, pitavastatin, and simvastatin (Zingel et al., 2021). As a water-soluble drug, rosuvastatin improved the performance of rats in neurobehavioral tests and reversed high-salt and cholesterol diet induced changes in oxidative biomarkers, which were due to its good affinity with Nrf2 (Husain et al., 2018a). Another published paper showed that rosuvastatin also has a high affinity for the active site of NF-κB. Thus, rosuvastatin counteracts high-salt and cholesterol diet induced neuroinflammation and cognitive impairment by reducing TNF-α and increasing IL-10 through inhibiting the overexpression of NF-κB in the hippocampus (Husain et al., 2017). Simvastatin, one of the most common lipid-soluble statins, may slow the progression of cognitive decline, as a conclusion from a reanalysis of patient-level data on AD obtained from failed clinical trials (Geifman et al., 2017). Simvastatin effectively reduced Aβ_42_ protein levels in yeast cells (Dhakal et al., 2019). In brain capillary endothelial cells, simvastatin treatment significantly increased intracellular apoJ levels. ApoJ can bind Aβ, thus promoting Aβ clearance through BBB and reducing Aβ uptake (Zandl-Lang et al., 2018). Although simvastatin readily passes through the BBB, a study suggests that free simvastatin failed to improve cognitive impairment caused by high-cholesterol diets and that it improves cognitive dysfunction only when simvastatin was reproduced in lipid-core nanocapsules (Lorenzoni et al., 2020). However, not all studies specify that statins can protect against cognitive deficits caused by hypercholesterolemia. Recent meta-analysis pointed out that contemporary lipid-lowering drugs such as statins did not show a significant difference with the incidence of cognitive impairment in randomized controlled trials (Xuan et al., 2020; Ying et al., 2021). And even, statins are harmful to cognitive function. The Food and Drug Administration has added an adverse event message to the labeling of statins that statins have the potential to cause reversible cognitive impairment (U.S. Food and Drug Administration, 2015). Inhibition of both protein farnesyltransferase (FT) and protein geranylgeranyltransferase-1 (GGT) by statins may provide an explanation for the inconsistent results in AD treatment. Heterozygous deletion of either FT or GGT reduces Aβ deposition and neuroinflammation, but only haplodeficiency of FT rescues cognitive function in APP/PS1 mice (Cheng et al., 2013). Of note, GTT is critical for synapse formation and remodeling. In GGT-haplodeficient mice, reduced dendritic spine density in cortical neurons and impaired hippocampal synaptic plasticity were observed (Hottman et al., 2018). Thus, the reduction in GGT by statins likely offsets the benefit of FT reduction. In addition to statins, drugs that block intestinal cholesterol absorption are also known to lower cholesterol levels. Ezetimibe is a drug that reduces cholesterol absorption and targets Niemann-Pick C1-like 1. In Swiss albino mice on a cholesterol diet, ezetimibe administration lowers cholesterol levels and improves memory (Dalla et al., 2009). However, ezetimibe increased enterocyte Aβ abundance in C57BL/6J mice, which may affect cerebral Aβ homeostasis (Pallebage-Gamarallage et al., 2009). Additionally, Niacin also has the ability to lower serum cholesterol, and a prospective study in Chicago suggests that niacin supplementation may prevent AD (Morris et al., 2004). Altogether, statins are widely studied, but no consistent conclusions have been reached in terms of AD prevention and treatment.

### Drugs Targeting ACAT, CYP46A1, LXR, RXR, and PPAR

ACAT is an intracellular membrane-bound enzyme responsible for converting cholesterol into cholesteryl esters (Rogers et al., 2015). Earlier work showed that ACAT inhibitors (CP-113,818 and CI-1011) reduced cholesterol esters in APP transgenic mice and reduced soluble Aβ_42_ and Aβ plaques in the brain (Hutter-Paier et al., 2004; Huttunen et al., 2010). In human H4 neuroglioma cells, knockdown of ACAT1 reduced the proteolytic processing of APP and Aβ production (Huttunen et al., 2007). In a different study, which applied ACAT1 knockdown gene therapy to AD mice, similar results were obtained (Murphy et al., 2013). ACAT1 gene ablation ameliorates cognitive deficits in AD mice, which may be attributed to improved Aβ pathology by increasing 24-OHC content (Bryleva et al., 2010). It is known that cholesterol 24-hydroxylation is catalyzed by CYP46A1, which can be activated by efavirenz at low concentrations. In 5XFAD mice, efavirenz treatment reduced amyloid abundance in the brain and decreased the total number and area of Aβ plaques (Mast et al., 2017). In another study, efavirenz treated 5XFAD mice increased 24-OHC levels and improved cognition but had no effect on amyloid plaque load (Petrov et al., 2019). 24-OHC is usually beneficial to the brain, whereas excess 27-OHC is probably involved in AD pathogenesis. 27-OHC is produced from cholesterol catalyzed by the enzyme CYP27A1. Unfortunately, studies of drugs targeting CYP27A1 are lacking. Besides esterification and conversion, cholesterol can be excreted from cells via ABC transport. ABCA1 transcription and APOE expression are regulated by LXR, RXR and PPAR, which have implications for cholesterol transport and Aβ clearance. T0901317 is widely studied as an agonist of LXR. T0901317-treated APP23 mice showed an increase in ABCA1 expression with a decrease in soluble Aβ _40_ and Aβ _42_ levels in the brain (Koldamova et al., 2005). Similar results were observed in APP23 mice on a high-fat diet (Fitz et al., 2010). Further study showed that T0901317 reduced interstitial fluid levels of Aβ but had no effect on already-formed Aβ plaques (Fitz et al., 2014). In Tg2576 mice, T0901317 promotes Aβ _42_ clearance but did not inhibit APP processing (Riddell et al., 2007). In APPLSxPS1mut mice and APP/E4 ABCA1 haplo-deficient (APP/E4/Abca1 ±) mice, T0901317 improved memory function, despite failing to reduce Aβ plaque load (Vanmierlo et al., 2011; Carter et al., 2017). A recent study indicates that T0901317 can antagonize Aβ-induced toxicity and exert a protective effect on human neural stem cells (Chiang et al., 2022). GW3965, another LXR agonist, improves synaptic function in primary hippocampal neurons exposed to Aβ (Báez-Becerra et al., 2018). In addition, GW3965 reduces Aβ deposition in APP/PS1 mice (Donkin et al., 2010). Since LXR activates gene transcription by forming a heterodimer with RXR (Baranowski, 2008), agonists targeting RXR have also been extensively studied. Bexarotene, an RXR agonist, has been reported to reduce Aβ plaque load, increase Aβ clearance, and improve cognitive function in APP/PS1 mice (Cramer et al., 2012). However, subsequent studies failed to confirm that bexarotene reduced Aβ plaque burden (Price et al., 2013; Tesseur et al., 2013; Veeraraghavalu et al., 2013). Another study suggests that bexarotene improved cognitive deficits in APP mice, although it did not affect Aβ (Fitz et al., 2013). However, no cognitive benefit of bexarotene in APP/PS1 mice has also been reported (LaClair et al., 2013). In 5XFAD mice, Bexarotene treatment reduced amyloid plaque accumulation but not Aβ_42_ (Mariani et al., 2017). PPARγ activation stimulates LXR expression, and significantly, PPARγ also plays a role in glucose regulation and inflammation inhibition (Landreth et al., 2008). However, the PPARγ activator rosiglitazone failed to improve cognitive performance in AD patients (Gold et al., 2010; Tzimopoulou et al., 2010).

### Herbal Medicine and Natural Compound

The cholesterol-lowering effects of herbs and natural compounds may reduce 27-OHC influx to the brain, as serum cholesterol levels have a positive correlation with 27-OHC levels (Hirayama et al., 2009). Safflower yellow, a flavonoid isolated from safflower, reduces endogenous cholesterol by decreasing the expression of mevalonate decarboxylase and APOE4 in the cortex, thereby improving learning and memory performance in AD mice (Du et al., 2021). Troxerutin is also a naturally occurring flavonoid that improves cognitive impairment induced by brain insulin resistance in mice fed with HCD (Lu et al., 2011). On the one hand, troxerutin reduces cholesterol levels by inhibiting the activation of c-jun N-terminal kinase 1 and IκB kinase β/NF-κB. On the other hand, troxerutin alleviates oxidative stress by reducing the levels of ROS, protein carbonyls and advanced glycation end products (Lu et al., 2011). Quercetin, a polyphenolic flavonoid, reverses HCD-induced cognitive deficits by lowering cholesterol levels, reducing Aβ levels, and decreasing oxidative stress and neuroinflammation (Lu et al., 2010). Additionally, cognitive impairment induced by HCD can be partially reversed by treatment with a tannins-enriched fraction of *Emblica officinalis*, which exerts its anti-inflammatory effects by inhibiting NF-κB nuclear translocation (Husain et al., 2018c) and exerts its antioxidant stress effects by activating the Nrf2-ARE pathway (Husain et al., 2018b). In addition to flavonoids, some other chemical components also possess biological activities to improve HCD-induced cognitive deficits. Phytosterol ester reduces serum lipid levels in HCD rats and improves cognitive performance by reducing neuroinflammation and improving the cholinergic system (Rui et al., 2017). Dill tablets and Ocimum basilicum L. attenuates cognitive dysfunction in rats by restoring histopathological changes and delaying Aβ accumulation, which was attributed to a decrease in serum cholesterol and a reduction in oxidative stress (Mohammadali et al., 2020; Heshami et al., 2021). Walnut polyphenols similarly improved memory function in hypercholesterolemic mice by lowering cholesterol and reducing oxidative stress (Shi et al., 2014). In addition, polyphenols from Oriental plums ameliorated cognitive decline in HCD mice, which mechanism involved lowering cholesterol and reducing BACE1 and Aβ expression (Kuo et al., 2015). Thymoquinone not only down-regulates BACE1 and RAGE levels to reduce the source of Aβ, but also up-regulates IDE and LRP1 levels to promote Aβ degradation and thus resist HCD-induced Aβ accumulation (Ismail N. et al., 2017). Royal jelly, a secretion with complex composition, regulates Aβ metabolism by a mechanism like thymoquinone and reduces cholesterol levels in HCD-fed rabbits (Pan et al., 2018). Similar results were observed in ovariectomized cholesterol-fed rabbits, where royal jelly treatment reduced lipid levels and Aβ aggregation, as well as increased cholinergic receptor activity and antioxidant capacity, which contributed to improved cognitive impairment (Pan et al., 2019). Overall, herbal extracts and natural compounds show promise to improve HCD-induced cognitive impairment, but more research is needed to investigate their effects on 27-OHC.

## Conclusion and Future Perspectives

Patients with hypercholesterolemia are at risk for developing AD, as data obtained from human and animal models suggest an association between peripheral cholesterol levels and AD development. Of note is that serum cholesterol is not allowed to enter the brain, whereas oxysterols can easily cross the BBB (Rhea and Banks, 2021). In AD patients, oxysterol levels are significantly changed, among which 24-OHC and 27-OHC are considered additional biomarkers for AD diagnosis (Wang et al., 2016). Generally, 24-OHC flows from the brain into the periphery, whereas 27-OHC flows from the periphery into the brain (Gamba et al., 2021). 27-OHC is positively correlated with plasma cholesterol levels (Burkard et al., 2007; Nelson et al., 2013), and plays an important role in AD pathogenesis. Hence, 27-OHC is likely to be a key link between hypercholesterolemia and AD. Statins are known for their cholesterol-lowering effects, but statins have had mixed results in preventing AD, and other cholesterol-lowering drugs are less well studied. Drugs targeting cholesterol esterification, conversion, and transport have mostly shown improvements in cognitive deficits in animal models, and herbs and natural compounds with cholesterol-lowering effects have also shown benefits. In terms of drug efficacy studies, there are limited studies directly targeting 27-OHC. Further research is needed to investigate the mechanisms involved in 27-OHC to answer whether targeting 27-OHC is an effective measure against AD. Additionally, the long-term effects of lipid management need to be further investigated, and cholesterol levels should be monitored from midlife to develop appropriate strategies for slowing AD progression.

## Author Contributions

MW, YZ, XL, and WC searched for relevant literature and wrote the manuscript content. RL, LM, YH, and DZ searched for relevant literature. YL, WZ, JF, SF, and YC revised the manuscript. QW and WL conceived the idea and specified the content. All authors contributed to the article and approved the submitted version.

## Conflict of Interest

The authors declare that the research was conducted in the absence of any commercial or financial relationships that could be construed as a potential conflict of interest.

## Publisher’s Note

All claims expressed in this article are solely those of the authors and do not necessarily represent those of their affiliated organizations, or those of the publisher, the editors and the reviewers. Any product that may be evaluated in this article, or claim that may be made by its manufacturer, is not guaranteed or endorsed by the publisher.
